# Mirogabalin inhibits scratching behavior of spontaneous model mouse of atopic dermatitis

**DOI:** 10.3389/fphar.2024.1382281

**Published:** 2024-06-26

**Authors:** Kosuke Matsuda, Yutaka Kitano, Masahito Sawahata, Toshiaki Kume, Daisuke Uta

**Affiliations:** ^1^ Department of Applied Pharmacology, Graduate School of Medicine and Pharmaceutical Sciences, University of Toyama, Sugitani, Toyama, Japan; ^2^ R&D Division, Daiichi Sankyo Co., Ltd., Tokyo, Japan

**Keywords:** mirogabalin, atopic dermatitis, chronic itch, α2δ-1 subunit, spinal dorsal horn, pregabalin

## Abstract

**Introduction:** Atopic dermatitis (AD) is one of the most prevalent intractable chronic itch diseases worldwide. In recent years, new molecular-targeted drugs have emerged, but side effects and economic challenges remain. Therefore, since it is important for AD patients to have a wider range of treatment options, it is important to explore new therapeutic agents. Gabapentinoids, gabapentin and pregabalin, have been shown to be effective for the clinical treatment of several chronic itch. Recently, mirogabalin (MGB) was developed as a novel gabapentinoid. MGB is a drug for neuropathic pain and has a margin of safety between its side effects and the analgesic effect for animal experiments. Herein, we showed that MGB exhibited an antipruritic effect in a mouse model of AD using NC/Nga mice.

**Methods and results:** The oral administration of MGB (10 mg/kg) inhibited spontaneous scratching behavior in AD mice and its effect was dose dependently. Then, when MGB (10 mg/kg) was orally administrated to healthy mice, it did not affect motor function, including locomotor activity, wheel activity, and coordinated movement. Moreover, gabapentin (100 mg/kg) and pregabalin (30 mg/kg), inhibited spontaneous scratching behavior in AD mice and decreased motor function in healthy mice. Furthermore, intracisternal injection of MGB (10 μg/site) significantly suppressed spontaneous scratching behavior in AD mice.

**Discussion:** In summary, our results suggest that MGB exerts an antipruritic effect via the spinal dorsal horn using NC/Nga mice. We hope that MGB is a candidate for a novel therapeutic agent for AD with relatively few side effects.

## 1 Introduction

Atopic dermatitis (AD) is a common chronic inflammatory dermatosis with persistent pruritus (Sroka-Tomaszewska and Trzeciak, 2021). Approximately 20% of children and 2%–7% of adults worldwide are affected by AD (Son et al., 2017). The pathophysiology of AD is complex and mainly involves a genetic predisposition and the loss of barrier function (Li et al., 2021). Additionally, scratching causes further inflammation (itch-scratch cycles), leading to chronic itch.

Depending on the country and region, the first-line drugs for AD treatment are topical corticosteroids (TCS) and topical calcineurin inhibitors that inhibit skin inflammation (Chiricozzi et al., 2020; Frazier and Bhardwaj, 2020). However, steroids pose safety concerns including the theoretical risk of systemic absorption of potent and ultrapotent agents, as well as thinning and atrophy of the sensitive skin. These problems are rare when appropriately used; however, steroid phobia among patients and caregivers limits adherence (Chovatiya and Paller, 2021). Alternatively, topical calcineurin inhibitors, including tacrolimus, are often used in combination with TCS, but their safety is questionable because of a possible link between topical calcineurin inhibitors and malignancy (Thaçi et al., 2008; Martins et al., 2015; Frazier and Bhardwaj, 2020). Recently, biologic therapies and Janus kinase (JAK) inhibitors have attracted considerable attention as novel therapeutic agents (Tameez Ud Din et al., 2020). Although they significantly improve the patient’s condition, there are concerns about side effects due to immunosuppression and high economic burden (Canadian Agency for Drugs and Technologies in Health, 2023; Lugović-Mihić et al., 2023). Therefore, it is expected to develop new therapeutic agents for AD that can be easily used by a variety of patients.

It is generally accepted that itch suppression is an important treatment strategy for AD (Hashimoto et al., 2004; Tominaga and Takamori, 2022). Scratching contributes significantly to the worsening of the pathology, and the itch itself is the most significant cause of reduced quality of life in patients with AD (Wahlgren, 1999). In a survey conducted mainly in the United States, as many as 89.8% of patients reported that reducing itch was the treatment goal (Schmitt et al., 2008). However, antihistamines that act on the periphery have no effect on pruritus in AD because many intrinsic factors cause itch in the skin other than histamine (Wahlgren et al., 1990; Umehara et al., 2021). For this reason, it is important to develop antipruritic drugs that act on the central nervous system (CNS).

Gabapentinoids, including gabapentin (GBP) and pregabalin (PGB), are reported to be effective against several chronic pruritic disorders in clinical practice (Martinelli-Boneschi et al., 2017; Hercz et al., 2020). Gabapentinoids are antiepileptic and analgesic drugs (Kremer et al., 2016). These are specific ligands for the α_2_δ-1 subunit of the voltage-gated calcium channel (VGCC) and have an analgesic effect by acting on the spinal dorsal horn (SDH), mainly on the α_2_δ-1 subunits in the presynaptic terminals of sensory neurons (Gee et al., 1996; Field et al., 2006; Matsuzawa et al., 2014). In addition, mirogabalin (MGB), a recently developed novel gabapentinoid, is a therapeutic drug for the treatment of neuropathic pain. Due to its high selectivity for the α_2_δ-1 subunit compared to PGB, there is a margin of safety between the CNS side effects and the analgesic effect of a dose, as seen in rats (Domon et al., 2018; Deeks, 2019; Kato et al., 2021; Kim et al., 2021). However, it is not known whether gabapentinoids including MGB, are effective against chronic itch in AD.

Here, we analyzed the effects of MGB using the NC/Nga (NC) mouse as a model of spontaneous AD, which is characterized by a good mimicry of the clinical pathology.

## 2 Materials and methods

### 2.1 Animals

Specific pathogen free (SPF) and conventional (CV) NC mice were purchased from Japan SLC, Inc. (Shizuoka, Japan). Male mice (8–30 weeks old) were housed at a temperature of 25°C ± 1°C with a 12 h light-dark cycle (light from 7:00–19:00) and water and food (CE-2, CLEA Japan, Inc., Tokyo, Japan) were provided *ad libitum*. All animal experiments were conducted according to relevant national and international guidelines contained in the “Act on Welfare and Management of Animals” (Ministry of Environment of Japan), and procedures used in the animal experiments were approved by the Committee for Animal Experiments at the University of Toyama (A2019PHA-12, A2019PHA-13, A2022PHA-2, A2023PHA-13). Efforts were made to minimize animal suffering and counts.

### 2.2 Drugs

MGB besylate (code number: DS-5565) and PGB were provided by Daiichi Sankyo Co., Ltd. (Tokyo, Japan). GBP was obtained from Tokyo Chemical Industry Co., Ltd. (Tokyo, Japan). The drugs were dissolved in distilled water, which was procured from Otsuka Pharmaceutical Factory, Inc (Tokushima, Japan). In this study, dose levels were presented to reflect those of the free form. When given perorally, the drugs were administered at a volume of 0.1 mL/10 g body weight. To act locally on the cervical spinal cord, where NC mice scratch most often, we conducted intracisternal injection. For intracisternal injection, the drugs were dissolved in saline (Otsuka Pharmaceutical Factory, Inc., Tokushima, Japan) and administered at a volume of 5 μL/site *via* a disposable 27-gauge needle attached to a Hamilton microsyringe (Hamilton Company, Nevada, United States) after the mice were lightly anesthetized using isoflurane (FUJIFILM Wako Pure Chemical Corp., Osaka, Japan). We chose the dose for intracisternal injection of MGB for reference previously described (Kitamura et al., 2014).

### 2.3 Behavioral tests

All behavioral tests were conducted during the light period (7:00–19:00). The scratching behavior was recorded using the SCLABA^®^-Next (Noveltec Inc., Kobe, Japan) real-time scratch counting system. The animals were placed in an acrylic cage (approximately 150 mm wide, 200 mm deep, and 350 mm high) for at least 30 min before scratching behavior was measured. Locomotor activity was measured from image data recorded by SCLABA^®^-Next.

The running wheel test was performed for 30 min using activity wheel (SW-20; Melquest Ltd., Toyama, Japan) (Akiyama et al., 2018). The number of revolutions of the wheel cage was determined. The animals were placed in a wheel cage and trained for at least 30 min/d for 3 days prior to testing.

The rotarod (Acceler Rota-Rod for mice 7,650; Ugo Basile, Italy) test was performed for reference previously described (Kayser and Christensen, 2000). The animals were placed on the rod, and a timer switch was simultaneously activated to rotate the rod from approximately 3.3 rpm to approximately 38 rpm for a maximum 5 min. The timer was stopped when the animals fell to the surface or performed three rotations while holding the rod. The animals were tested for two sessions per day with a resting period of approximately 30 min between sessions. The animals were trained 3 min/d for 3 days before the test day. On the test day, only the animals that remained balanced on the rotating rod for 3 min (cut-off time) were selected for testing.

### 2.4 Tissue preparation

Mice were anesthetized with intraperitoneal injection of a mixture of three anesthetic agents: 0.75 mg/kg medetomidine hydrochloride (Domitor, Nippon Zenyaku Kogyo, Koriyama, Japan), 4.0 mg/kg midazolam (Midazolam (SANDOZ), Sandoz K.K., Tokyo, Japan), and 5.0 mg/kg butorphanol tartrate (Betorphal, Meiji Seika Pharma, Tokyo, Japan) and perfused transcardially with 10 mL phosphate-buffered saline (PBS) at pH 7.4, followed by 10 mL of 4% paraformaldehyde (Hiramatsu et al., 2021). The spinal cord (cervical1-5) was removed, immersed in the same fixative overnight, and then immersed in 25% sucrose in PBS for 48 h at 4°C. Tissues were cut into 30 μm thick sections by use of a cryostat (CM3050 S; Leica, Germany). The sections were then rinsed three times with PBS for 5 min each.

### 2.5 Immunohistochemistry

These methods were refined from previous reports (Takanami et al., 2010). For antigen activation, spinal cord sections were treated with HistoVT One (Nacalai Tesque, Kyoto, Japan) for 20 min at 70°C in PBS. The sections were then rinsed three times with PBS containing 0.3% Triton X-100 (PBS-T, Wako, Osaka, Japan) for 5 min each. Nonspecific binding components were blocked with 1% normal goat serum and 1% bovine serum albumin in PBS-T for 1 h at room temperature. Subsequently, the sections were treated with an antibody against α_2_δ-1 subunit (1:1,000; C5105; Sigma) in the blocking buffer for 2 days at 4°C. The sections were rinsed with PBS-T three times for 5 min each and treated for 120 min at room temperature with Alexa Fluor 488-labeled anti-rabbit IgG (1:1,000, Thermo Fisher Scientific, Waltham, MA, United States). Sections were rinsed with PBS-T, and mounted on glass slides (Matsunami Glass, Kishiwada, Japan), and coverslipped (Matsunami Glass) with mounting medium (FLUOROSHIELD; ImmunoBioScience Corp, CA, United States). Fluorescence images were captured using a microscope (BZ-X800; Keyence Corporation, Osaka, Japan).

### 2.6 Statistical analysis

Results are expressed as the mean ± standard error of the mean. Statistical differences between the two groups for various behavior tests were analyzed using the unpaired *t*-test and Mann Whitney test. If the normality test or equal variance test failed, the unpaired *t*-test was replaced with the Mann Whitney test. Multiple-group comparisons were performed using a one way analysis of variance (ANOVA) followed by the Tukey’s test and Dunnett’s test, and Kruskal–Wallis test followed by Dunnett’s test. All tests were considered statistically significant at *p* < 0.05. Prism 5 (GraphPad Software Inc., La Jolla, CA, United States) was used for the statistical analyses.

## 3 Results

### 3.1 Analysis of onset of AD in CV mice

**FIGURE 1:**
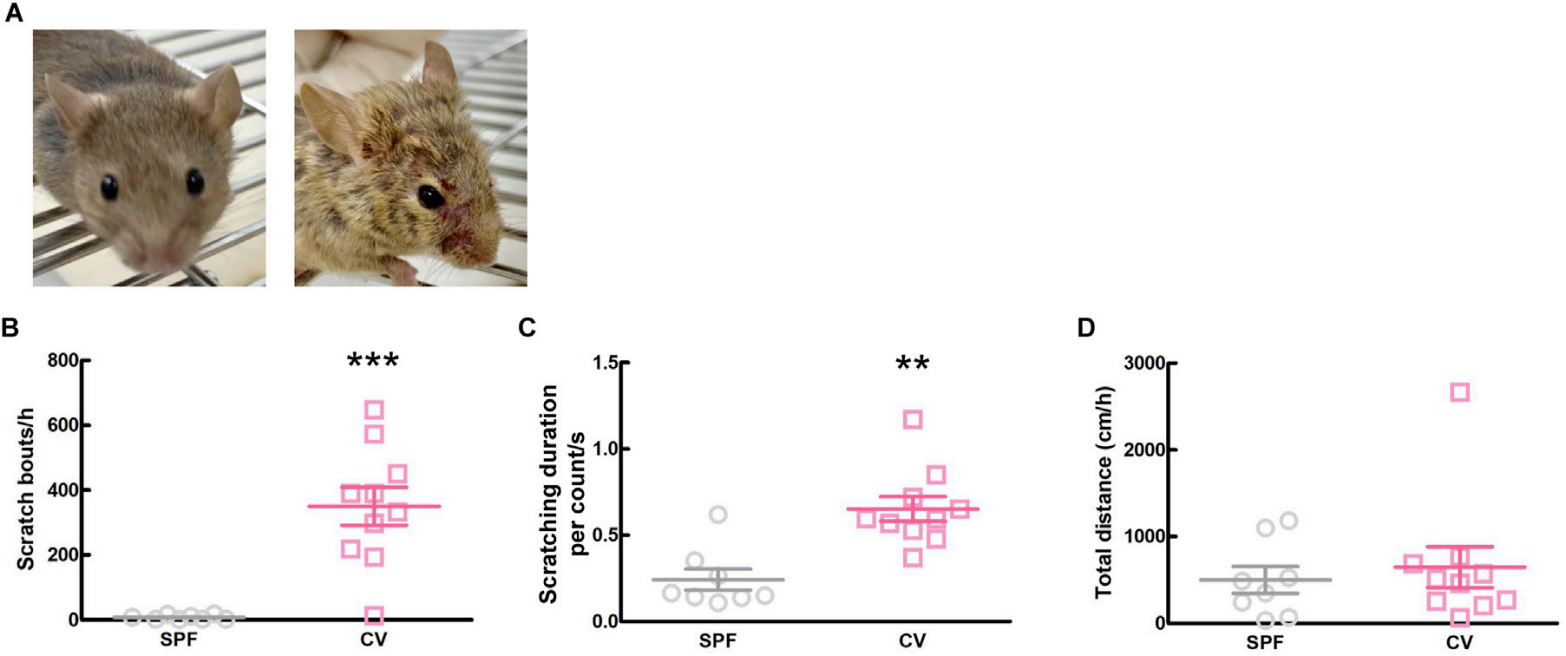
The comparisons of dermatitis and spontaneous behavior between SPF and CV mice. **(A)** Representative images of inflammation of face in SPF (*left*) and CV (*right*) mice. **(B–D)** Behavioral analysis between SPF and CV mice. Values represented the means and S.E.M (*n* = 8–10). **(B)** The number of scratching behaviors for 1 h ***p < 0.001, (Mann Whitney test). **(C)** Duration for one scratching behavior. **p < 0.01, (Mann Whitney test). **(D)** Total distance moved for 1 h as locomotor activity.

Comparisons between healthy SPF and CV mice with AD are shown in Figure 1. Dermatitis was observed all over the body, including the face, in CV mice compared to SPF mice (Figure 1A). There was a significant difference in scratching bouts within 1 h between the CV and SPF groups (*p* < 0.001, Figure 1B). The scratching duration per scratching was significantly longer in the CV group than in the SPF group (*p* = 0.003, Figure 1C). Moreover, there was no difference in the total distance moved between the SPF and CV group (Figure 1D).

### 3.2 Effects of MGB on spontaneous scratching behavior in CV mice

**FIGURE 2:**
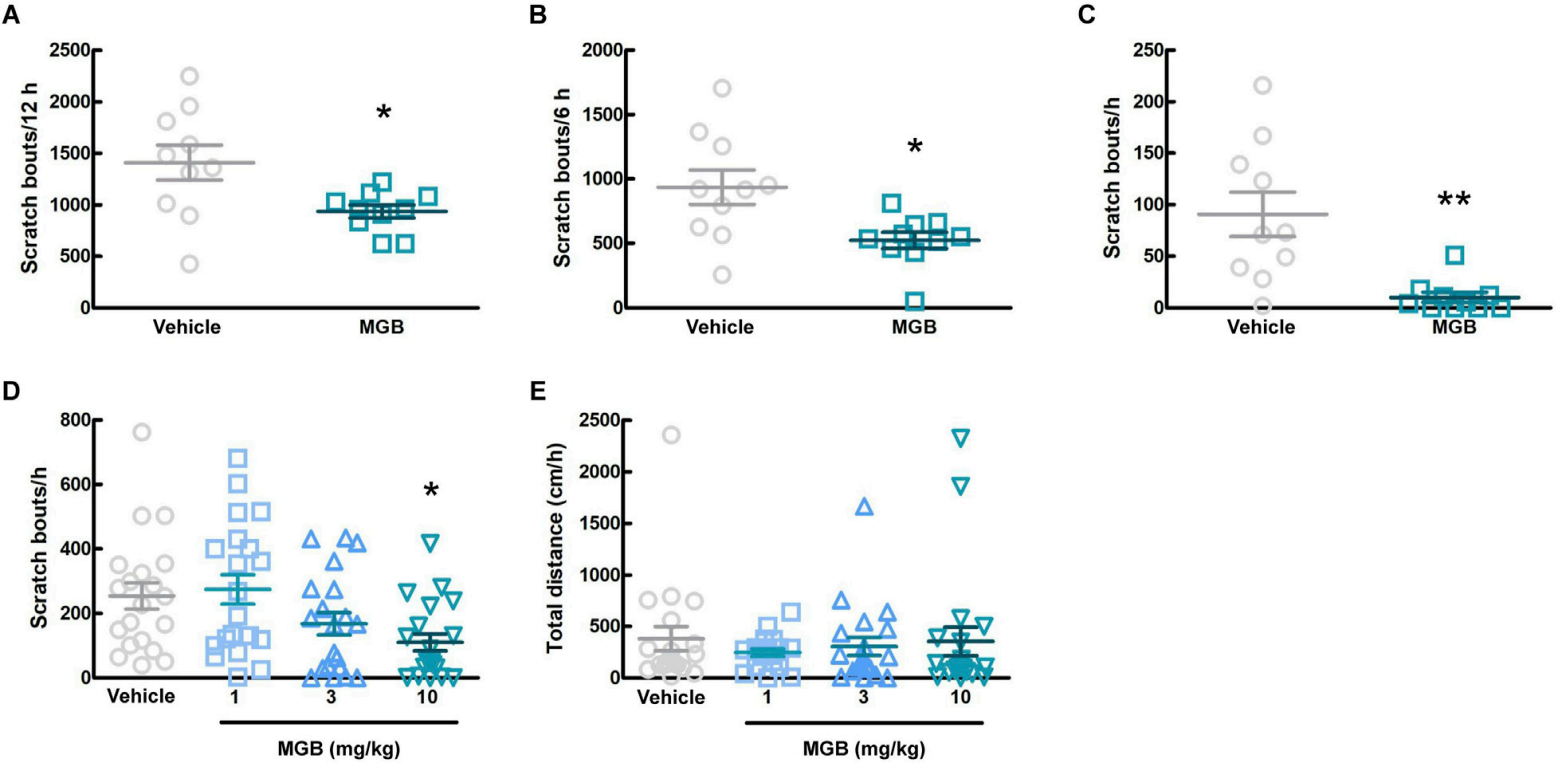
Effect of mirogabalin (MGB) on scratching behavior and locomotor activity in CV mice. **(A–C)** The number of spontaneous scratching behavior of CV mice was measured for 12 h after oral administration of vehicle or MGB (10 mg/kg). Values represented the means and S.E.M (*n* = 10). **(A)** Analyzed for 12 h *p < 0.05, (Mann Whitney test). **(B)** Analyzed for 6 h *p < 0.05, (Unpaired *t*-test). **(C)** Analyzed for 1 h from 2.5 to 3.5 h after administration. **p < 0.01, (Mann Whitney test). **(D, E)** Dose-dependent effect of MGB in CV mice. Values represented the means and S.E.M (*n* = 20). **(D)** The number of scratching behavior for 1 h *p < 0.05, (vs. vehicle, Kruskal–Wallis test–Dunnett’s test). **(E)** Total distance moved for 1 h as locomotor activity.

We investigated whether MGB has an antipruritic effect and its action time. When the vehicle (distilled water) or MGB (10 mg/kg) was administered orally to CV mice and analyzed for 12 h, MGB produced an antipruritic effect (*p* = 0.031, Figure 2A; Supplementary Figure S1). In addition, when we analyzed chronologically, MGB had the effect for up to 6 h after administration (*p* = 0.012, Figure 2B). Further analysis indicated that the antipruritic effect of MGB appeared to be present at least between 2.5 and 3.5 h (*p* = 0.002, Figure 2C). Thereafter, we decided that behavioral experiments were conducted 2.5–3.5 h after the administration of MGB. When the vehicle or MGB (1, three or 10 mg/kg) was administered orally, MGB produced the antipruritic effect in a dose-dependent manner (vehicle vs. 10 mg/kg, *p* < 0.05, Figure 2D). Additionally, locomotor activity among these four groups did not show a significantly change (Figure 2E).

### 3.3 Effects of MGB on motor function in SPF mice

**FIGURE 3:**
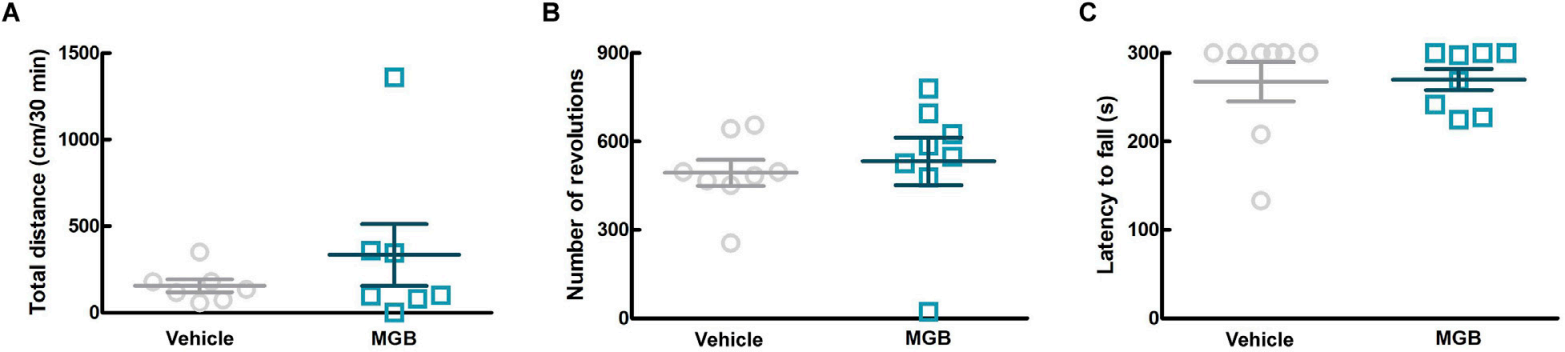
Effect of MGB on locomotor function in SPF mice. **(A–C)** Vehicle or MGB (10 mg/kg) were administered orally in SPF mice before behavioral test. Values represented the means and S.E.M (*n* = 7–8). **(A)** Locomotor activity test. **(B)** Running wheel test. **(C)** Rotarod test.

Gabapentinoids are associated with the sedation as a side effect (Gou et al., 2021). In order to investigate more details of the effect of MGB on motor function, we conducted three behavioral tests, namely, the rotarod test, the running wheel test, and the locomotor activity test (using SCLABA^®^-Next) in SPF mice. Oral administration of the vehicle or MGB (10 mg/kg) caused no significant changes (Figure 3). These results suggest that MGB (10 mg/kg) does not have a sedative effect.

### 3.4 Effects of other gabapentinoids on spontaneous scratching behavior in CV mice

**FIGURE 4:**
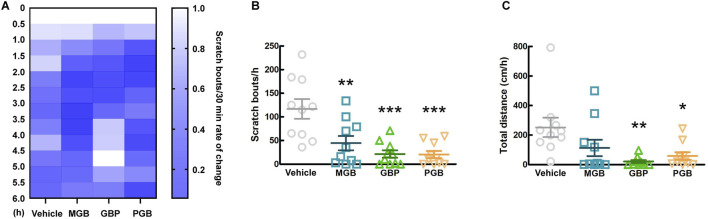
Effect of gabapentinoids on scratching behavior and locomotor activity in CV mice. **(A–C)** Vehicle, MGB (10 mg/kg), gabapentin (GBP, 100 mg/kg) or pregabalin (PGB, 30 mg/kg) were administered orally in CV mice. Values represented the means and S.E.M (*n* = 10). **(A)** The heatmap represents rate of temporal change of scratching behaviors, normalized by the number up to 0–0.5 h. **(B)** The number of scratching behaviors for 1 h from 1.5 to 2.5 h ***p < 0.001, **p < 0.01, (vs. vehicle, One Way ANOVA-Tukey’s test). **(C)** Total distance moved for 1 h as locomotor activity. **p < 0.01, *p < 0.05, (vs. vehicle, One Way ANOVA-Tukey’s test).

The conventional gabapentinoids, GBP and PGB, were originally developed as anticonvulsants, and then currently are indicated for chronic pain disorders, such as neuropathic pain as well (Kremer et al., 2016). First, we investigated the antipruritic effects of GBP, PGB, and MGB and the duration of their activity. We orally administered the vehicle, MGB (10 mg/kg), GBP (100 mg/kg), or PGB (30 mg/kg) to CV mice and monitored them for 6 h. Figure 4A shows the chronological rate of change in the scratch bouts as a heatmap. Between 1.5 and 2.5 h after administration, differences were observed in the MGB, GBP, and PGB groups compared to the vehicle group. Therefore, we decided to further analyze this period. Compared with the vehicle group, all gabapentinoids showed significantly suppressed scratch bouts (vs. vehicle group, *p* < 0.05, Figure 4B). However, we observed a significant decrease in locomotor activity in GBP and PGB groups (vs. vehicle group, *p* < 0.05, Figure 4C). This indicates that GBP and PGB may have sedative effects.

### 3.5 Effects of other gabapentinoids on motor function in SPF mice

**FIGURE 5:**
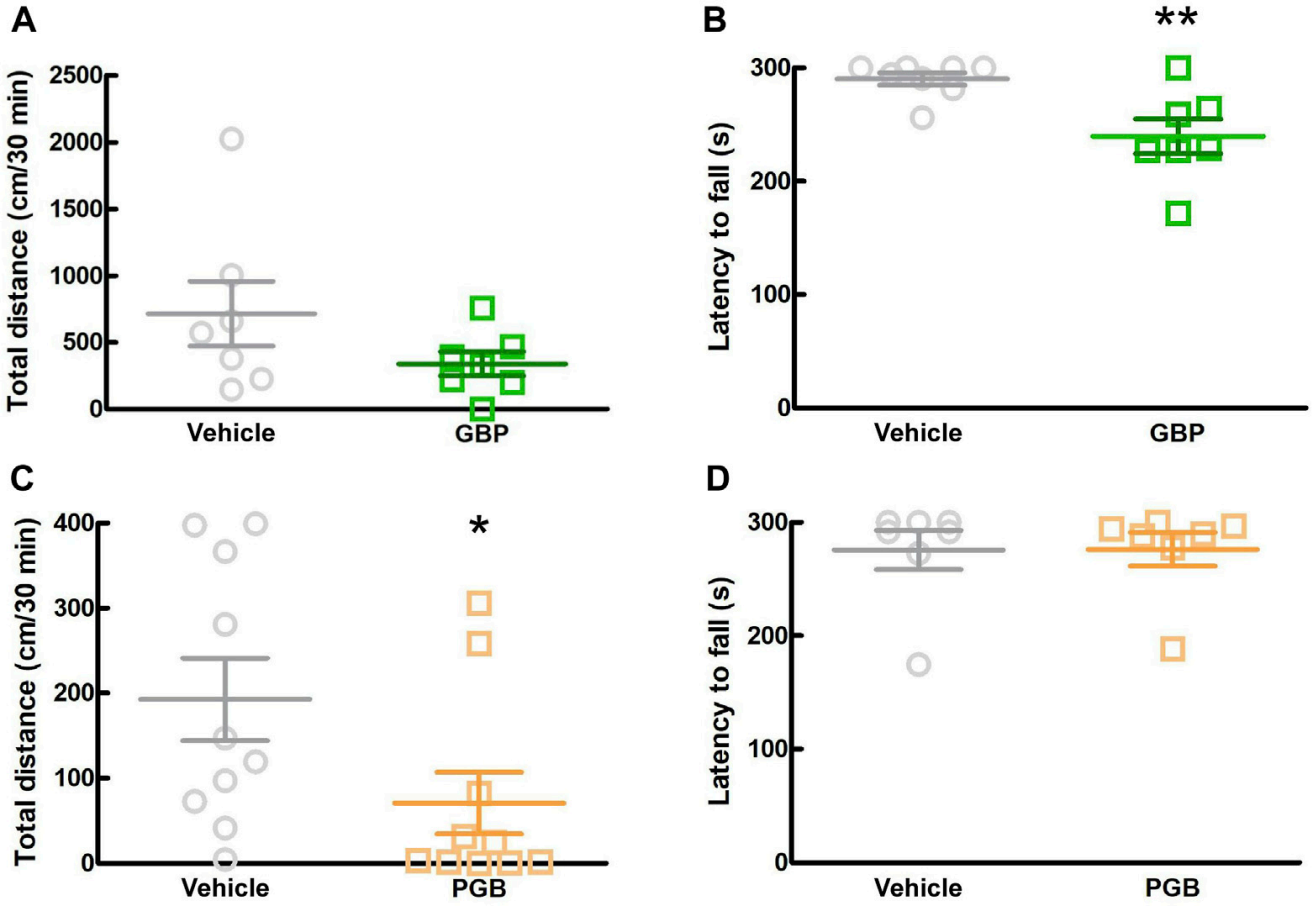
Effect of GBP and PGB on locomotor function in SPF mice. **(A, B)** Vehicle or GBP (100 mg/kg) were administered orally in SPF-mice. Values represented the means and S.E.M (*n* = 6–8). **(A)** Locomotor activity test. **(B)** Rotarod test. **p < 0.01, (vs. vehicle, Unpaired *t*-test). **(C, D)** Vehicle or PGB (30 mg/kg) were administered orally in SPF-mice. Values represented the means and S.E.M (*n* = 5–10). **(C)** Locomotor activity test. *p < 0.05, (vs. vehicle, Mann Whitney test). **(D)** Rotarod test.

In this study, we investigated the sedative effects of GBP and PGB in SPF mice. We conducted two behavioral tests, the locomotor activity test and rotarod test between 1.5 and 2.5 h after administration. In the locomotor activity test, GBP did not affect the total distance moved by SPF mice, whereas in the rotarod test, it significantly reduced the latency to fall (Figure 5A; *p* = 0.006; Figure 5B). In contrast, PGB decreased the total distance moved, but did not affect the latency to fall. (*p* = 0.017, Figures 5C, D). These data suggest that GBP and PGB exert sedative effects.

### 3.6 Analysis of the action sites of MGB

**FIGURE 6:**
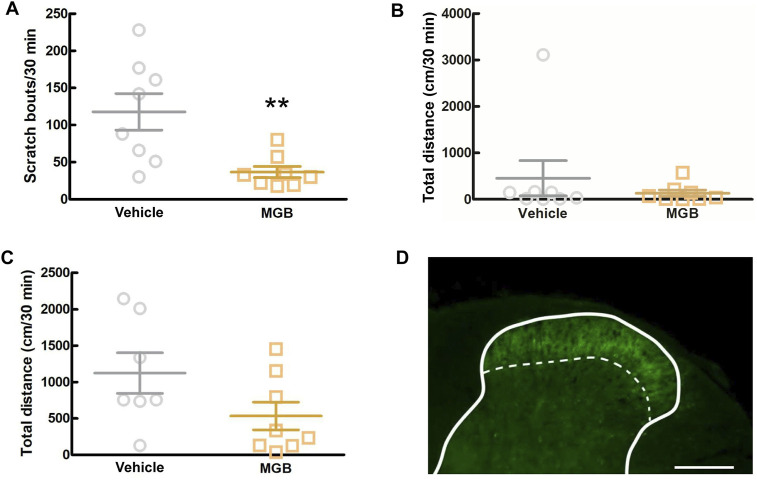
Analysis of the action target of MGB. **(A, B)** Effect of intracisternal injection of MGB in CV mice. Values represented the means and S.E.M (*n* = 7–8). **(A)** Scratching behavior of CV mice was analyzed after saline or MGB (10 µg) were administered. **p < 0.01, (vs. vehicle, Mann Whitney test). **(B)** Locomotor activity of CV mice was analyzed after saline or MGB (10 µg) were administered. **(C)** Locomotor activity of SPF mice was analyzed after saline or MGB (10 µg) were administered. **(D)** Distribution of α_2_δ-1 subunit in spinal dorsal horn. The spinal dorsal horn sections of CV mice were stained with anti-α_2_δ-1 antibody (green). Scale bar = 200 µm. Solid line represents the gray matter border, dotted line represents the superficial layer.

To elucidate whether MGB exerts an antipruritic effect via the CNS, including the SDH, we intracisternally administered MGB (10 μg/site) to CV mice and measured the number of spontaneous scratching behavior for 6 h (Supplementary Figure S2). When we analyzed from 0.5 to 1.0 h after intracisternal administration, MGB (10 µg/site) significantly inhibited spontaneous scratching bouts compared to the saline group (*p* = 0.007, Figure 6A) and there was no significant change in locomotor activity (Figure 6B). Then, we performed the locomotor activity test using SPF mice during this period. When SPF mice were intracisternally injected with MGB (10 µg/site) and subjected to the locomotor activity test, no difference was observed between the saline and MGB groups (Figure 6C). The α_2_δ-1 subunit of VGCC, the target of MGB, is expressed at primary afferent fiber terminals in the SDH. Here, we used immunohistochemical staining to analyze the expression of the α_2_δ-1 subunit in the SDH. We observed the α_2_δ-1 subunit expression in the superficial layer of the SDH in CV mice (Figure 6D; Supplementary Figure S3). These data indicate that MGB exerts an antipruritic effect *via* the α_2_δ-1 subunit in the SDH.

## 4 Discussion

NC/Nga (NC) mice are the first reported mouse model for atopic dermatitis (AD) (Matsuda et al., 1997). They remain healthy under specific pathogen free (SPF) conditions, but spontaneously develop AD under conventional conditions (CV) when infected with mites (Jin et al., 2009). NC mice are suitable for behavioral pharmacological experiments to investigate antipruritic effects, because the environment for AD onset and its pathology closely mimics human AD. In this study, we confirmed the development of chronic itch in CV mice as the gross pathology of dermatitis, and spontaneous scratching behavior was consistent with previous reports (Matsuda et al., 1997). A single oral administration of mirogabalin (MGB) (10 mg/kg) significantly inhibited spontaneous scratching in the CV mice in a dose-dependent manner. MGB (10 mg/kg) has an analgesic effect (Domon et al., 2018). In addition, there are clinical reports of successful treatment of neuropathic itch-associated prurigo nodules at doses used in chronic pain (Okuno et al., 2021). Considering these findings, the antipruritic effect of this dose was reasonable. Gabapentinoids exhibit sedation as a side effect (Gou et al., 2021). However, MGB had no effect on motor function in SPF mice. These results indicate that MGB (10 mg/kg) does not have a sedative effect and that the suppression of spontaneous scratching behavior by MGB is not due to sedative effects. This report is the first to suggest the potential of a pharmacotherapeutic intervention using the novel gabapentinoid, MGB for the treatment of AD pruritus in NC mice.

In a previous study, conventional gabapentinoids, gabapentin (GBP) and pregabalin (PGB), suppressed oxazolone-induced chronic itch in mice (Tsukumo et al., 2011). Moreover, GBP and PGB are reported to be effective against several chronic pruritic disorders in clinical practice, but their use in for AD has not been reported (Martinelli-Boneschi et al., 2017; Hercz et al., 2020). In this study, similar to MGB, GBP (100 mg/kg) and PGB (30 mg/kg) suppressed spontaneous scratching in CV mice. This suggests that all gabapentinoids have antipruritic effects in AD. These doses have shown analgesic effects in animal models (Kiso et al., 2008; Atwal et al., 2019; Gou et al., 2021). However, GBP (100 mg/kg) in the rotarod test and PGB (30 mg/kg) in the locomotor activity test decreased the motor function in SPF mice. These results indicated the sedative effects of GBP and PGB. In previous study, GBP and PGB have sedative effects at higher doses (Kiso et al., 2008; Gou et al., 2021). Furthermore, 10 mg/kg or higher doses of MGB decreased locomotor function in rats, but MGB has a wider safety margin for central nervous system (CNS) side effects than PGB (Domon et al., 2018). These previous reports are consistent with the findings of the present study that at antipruritic doses, MGB did not have a sedative effect, whereas PGB did.

In this study, intracisternal administration of MGB decreased the number of spontaneous scratching behaviors without affecting locomotor function. In addition, immunohistochemical staining showed that the α_2_δ-1 subunits were expressed on the superficial layer of the spinal dorsal horn (SDH) in CV mice as well as previous report (Taylor and Garrido, 2008). Generally, the mechanism of analgesic effects of MGB is explained by its action on the α_2_δ-1 subunit at the presynaptic terminal of the C fiber in the SDH neurons, which then inhibits the influx of calcium ions and release of glutamate (Stahl et al., 2013; Kitano et al., 2019). Intrathecal injection of MGB in a mouse model of neuropathic pain significantly suppressed mechanical allodynia, whereas in a rat model of inflammatory pain induced by formalin, it significantly suppressed flinches (Komatsu et al., 2021; Oyama et al., 2021). Recently, in patch clamp recordings using spinal cord slices from a mouse model of peripheral nerve injury, MGB reduced the amplitude of EPSCs evoked by electrical stimulation of the deep layer in the SDH (Koga et al., 2023). In addition, nociceptive and itch information generated in the periphery enters the SDH primarily *via* C fibers, and these pathways often overlap (Dhand and Aminoff, 2014). Additionally, in the mouse dorsal root ganglion (DRG), 85% of α_2_δ-1 subunit-positive-DRG neurons showed transient receptor potential vanilloid-1 immunoreactivity (Akiyama et al., 2018). Considering these findings, it is suggested that MGB acts on the α_2_δ-1 subunits in the superficial layer in the SDH neurons and inhibits the signal of chronic itch by suppressing glutamate release from synaptic terminals.

The antipruritic effect of oral administration of MGB suggests that this effect is not only related to the SDH but also to the upper CNS. There are several descending pathways that project to the SDH via the locus coeruleus (LC) and others as mechanisms to suppress itch from the upper CNS (Nguyen et al., 2023). In previous study, intracerebroventricular injection of MGB suppressed mechanical allodynia in a neuropathic pain mouse model. This suppression was cancelled when yohimbine was administered concurrently to suppress the descending pathway (Oyama et al., 2021). Moreover, injection of GBP into the LC inhibits mechanical allodynia in rat models of neuropathic pain by activating the noradrenergic descending pathway (Hayashida et al., 2008). However, intracerebroventricular injection of MGB in an inflammatory rat model does not suppress flinches (Komatsu et al., 2021). Further studies are required to determine whether MGB acts the upper CNS.

However, whether MGB improves AD symptoms other than pruritus, remains unclear. Further studies are needed to determine whether the long-term suppression of spontaneous scratching behavior by repetitive administration of MGB can improve dermatitis and epidermal thickening, which are the main symptoms of AD.

In conclusion, MGB showed an antipruritic effect in a mouse model of AD by acting on the CNS directly, without immunosuppression. Moreover, this is the first report on the use of MGB and other gabapentinoids in NC mice, as a mouse model of AD that mimics the clinical environment of ticks and causes AD. We hope that MGB can be developed as a new antipruritic agent for AD with relatively few side effects.

## Data Availability

The datasets presented in this study can be found in online repositories. The names of the repository/repositories and accession number(s) can be found in the article/Supplementary Material.

## Abbreviations

AD, atopic dermatitis; CNS, central nervous system; CV, conventional; DRG, dorsal root ganglion; GBP, gabapentin; JAK, Janus kinase; LC, locus coeruleus; MGB, mirogabalin; NC, NC/Nga; PGB, pregabalin; SDH, spinal dorsal horn; SPF, specific pathogen free; TCS, topical corticosteroids; VGCC, voltage-gated calcium channel.
