# Supplementary material for: Mirogabalin inhibits scratching behavior of spontaneous model mouse of atopic dermatitis
Source: Front Pharmacol. 2024 Jun 26;15:1382281. doi: 10.3389/fphar.2024.1382281 (PMC11234176; doi:10.3389/fphar.2024.1382281)
Supplement: Supplementary file 1 [file Presentation1.PPTX]

## Slide 1
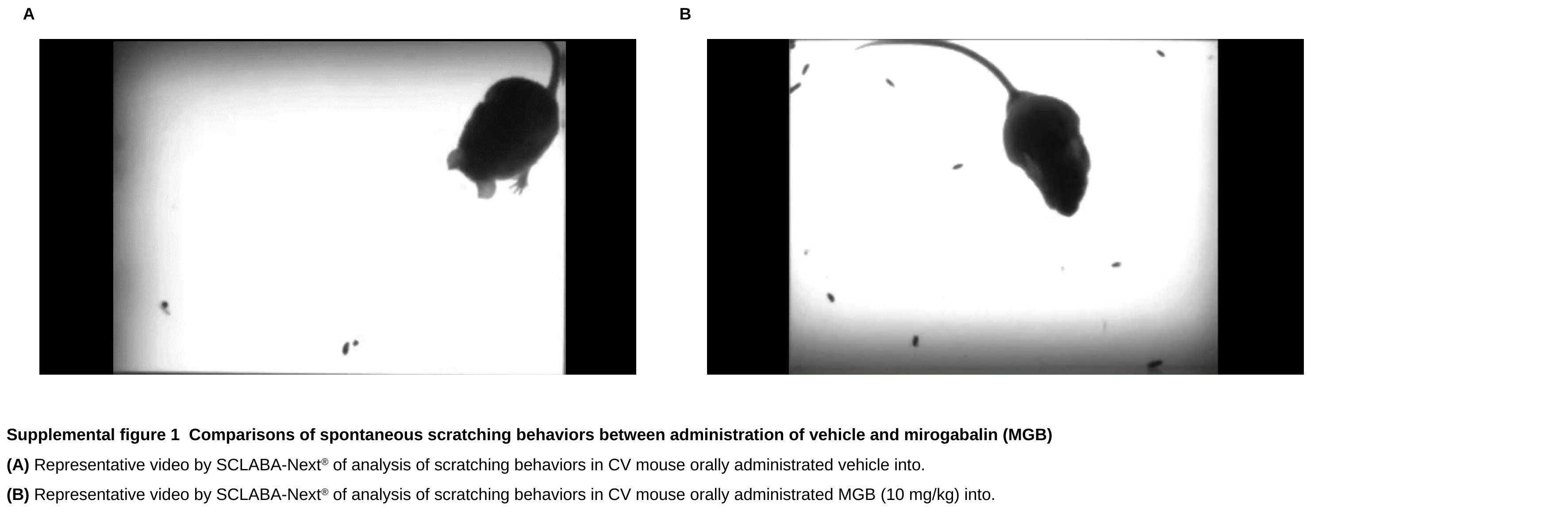

A
B
Supplemental figure 1 Comparisons of spontaneous scratching behaviors between administration of vehicle and mirogabalin (MGB)
(A) Representative video by SCLABA-Next® of analysis of scratching behaviors in CV mouse orally administrated vehicle into.
(B) Representative video by SCLABA-Next® of analysis of scratching behaviors in CV mouse orally administrated MGB (10 mg/kg) into.

## Slide 2
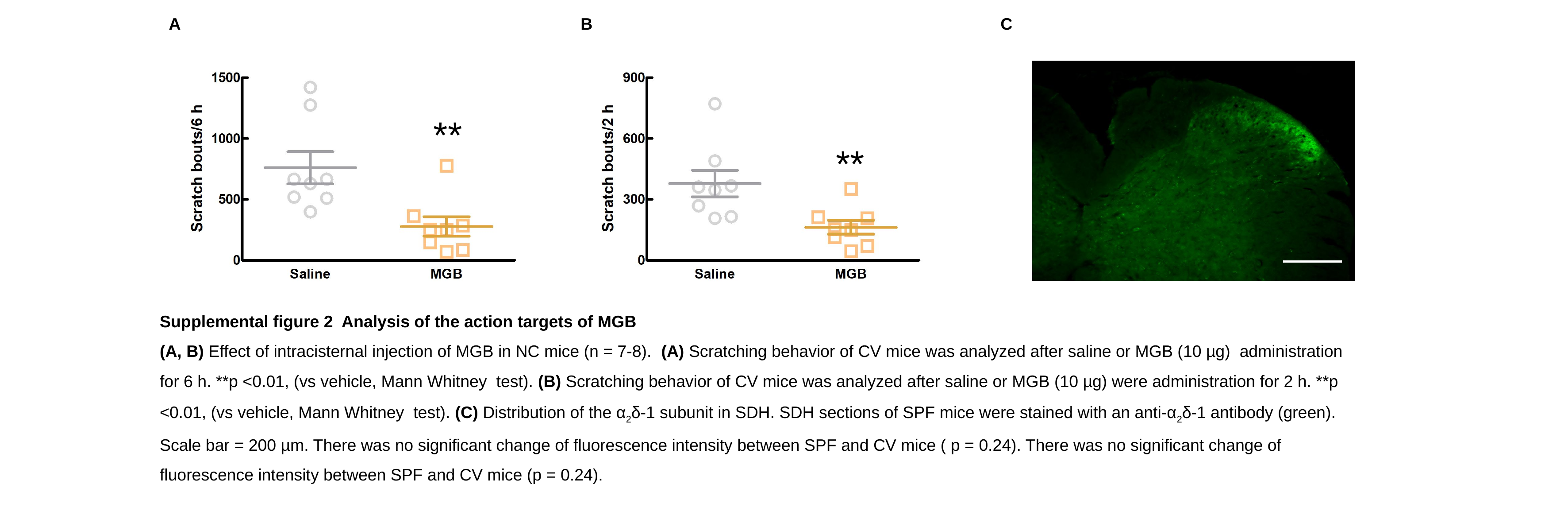

A
B
C
**
**
Supplemental figure 2 Analysis of the action targets of MGB
(A, B) Effect of intracisternal injection of MGB in NC mice (n = 7-8). (A) Scratching behavior of CV mice was analyzed after saline or MGB (10 µg) administration for 6 h. **p <0.01, (vs vehicle, Mann Whitney test). (B) Scratching behavior of CV mice was analyzed after saline or MGB (10 µg) were administration for 2 h. **p <0.01, (vs vehicle, Mann Whitney test). (C) Distribution of the α2δ-1 subunit in SDH. SDH sections of SPF mice were stained with an anti-α2δ-1 antibody (green). Scale bar = 200 µm. There was no significant change of fluorescence intensity between SPF and CV mice ( p = 0.24). There was no significant change of fluorescence intensity between SPF and CV mice (p = 0.24).
